# Comparison of diverting colostomy and fecal management catheter in fournier gangrene: a retrospective cohort study

**DOI:** 10.3389/fsurg.2026.1793103

**Published:** 2026-03-18

**Authors:** Mehmet Karahan, Cemal Hacıalioğlu, Selçuk Kaya, Metin Kement, Ozan Korkmaz, Sedef Poşul, Fırat Çağlar Sezmiş, Kerem Talha Düz, Alperen Gündüz, Hasan Kucuk

**Affiliations:** 1 General Surgery Department, Istanbul Kartal Dr. Lutfi Kirdar Education and Research Hospital, Istanbul, Türkiye; 2 General Surgery Department, Bahcesehir Universitesi, Beşiktaş, Türkiye

**Keywords:** colostomy, fecal diversion, fecal management catheter, fournier gangrene, necrotizing fasciitis

## Abstract

**Objective:**

This study aimed to compare the effectiveness of colostomy and fecal management catheterization, two fecal diversion methods, in the treatment of Fournier gangrene (FG) and to determine which method offers greater advantages.

**Materials and methods:**

A retrospective study was conducted on 54 patients who underwent surgical debridement and fecal diversion for FG at our hospital's General Surgery Clinic between January 2014 and October 2024. Patients were divided into colostomy (*n* = 28) and fecal management catheter (Flexi-Seal) (*n* = 26) groups for comparison. The primary outcome was in-hospital mortality; secondary outcomes were length of hospital stay, number of debridements, FGSI, and LRINEC scores. Covariate-adjusted analyses (ANCOVA and logistic regression) were performed to control for age and FGSI score differences between groups.

**Results:**

The mean age was 65.07 ± 13.45 years in the colostomy group and 57.92 ± 14.41 years in the Flexi-Seal group (*p* = 0.100). The mean number of debridements was 3.18 ± 2.58 in the colostomy group and 2.54 ± 1.56 in the Flexi-Seal group (*p* = 0.539). The mean length of hospital stay was 25.96 ± 16.02 days in the colostomy group and 20.50 ± 11.15 days in the Flexi-Seal group (*p* = 0.191). FGSI scores were 5.04 ± 3.44 and 3.88 ± 2.72 (*p* = 0.216), while LRINEC scores were 7.46 ± 2.24 and 7.00 ± 1.65 (*p* = 0.167) in the colostomy and Flexi-Seal groups, respectively. The mortality rate was 17.8% (5/28) in the colostomy group and 11.5% (3/26) in the Flexi-Seal group (*p* = 0.51; unadjusted OR 1.67; age- and FGSI-adjusted OR 0.95). The overall mortality rate was 14.8%. After covariate adjustment, the length-of-stay difference narrowed from 5.5 to 2.3 days, and the debridement difference from 0.64 to 0.43—all remaining non-significant. No statistically significant differences were observed between the groups regarding gender distribution and prevalence of comorbid diseases.

**Conclusion:**

Both fecal diversion methods demonstrated comparable effectiveness in FG treatment. After adjusting for age and FGSI score, the observed differences in length of hospital stay, debridement number, and mortality were substantially attenuated—with adjusted mortality OR approaching 1.0 (0.95)—confirming that the diversion method was not an independent predictor of outcomes. The numerically higher mortality in the colostomy group was explained by older patient age and higher disease severity. Both methods can be safely employed; patient-specific factors and clinical experience should guide method selection.

## Introduction

Fournier gangrene (FG) is a rapidly progressive and potentially fatal form of necrotizing fasciitis involving the perineal, genital, and perianal regions (1). It is characterized by synergistic polymicrobial infection, extensive fascial necrosis, and systemic inflammatory response, often leading to sepsis and multiple organ failure if not treated promptly. The incidence of the disease is reported as 1.6 per 100,000 people per year, and its frequency tends to increase with the rising prevalence of risk factors such as diabetes mellitus and immunosuppression (2, 3).

The etiology of the disease is attributed to anorectal (50%), urogenital, and cutaneous sources. Anorectal-derived FG is the most common type and is associated with a worse prognosis (1, 2). The clinical picture is initially characterized by nonspecific symptoms, and early diagnosis may be challenging. As the disease progresses, significant signs such as discoloration, cyanosis, and necrosis develop, and the risk of septic shock and multiple organ failure increases (4, 5).

The key components of FG treatment are early diagnosis, aggressive surgical debridement, broad-spectrum antibiotic therapy, and supportive care (6, 7). Surgical debridement involves removing all necrotic tissue and non-viable fascia until healthy tissue is reached. An average of 2–4 debridement sessions are reported in the literature (8, 9). The FGSI and LRINEC scoring systems are used for prognostic evaluation (10, 11).

One of the important and controversial issues in FG treatment is the need for fecal diversion. Particularly in cases of anorectal origin or extensive perianal involvement, protecting the wound from fecal contamination is critical for infection control and wound healing (12, 13). The most common method for fecal diversion is colostomy, but in recent years fecal management catheters have emerged as an alternative (14, 15). While colostomy provides effective fecal diversion, it carries disadvantages such as stoma complications and the need for closure surgery. Fecal management catheters, on the other hand, are less invasive but have limitations including catheter displacement and inadequate diversion (3, 16).

Studies comparing these two methods are limited in the literature. Therefore, in this study, we aimed to retrospectively examine patients treated for FG in whom fecal diversion was already clinically indicated, and to compare the effectiveness, safety, and outcomes of two diversion methods—colostomy and fecal management catheter—in terms of primary outcome (in-hospital mortality) and secondary outcomes (length of hospital stay, number of debridement procedures, and prognostic scores). Our focus is specifically the comparative question—which method performs better in patients for whom diversion has already been decided—rather than the broader question of whether fecal diversion is necessary.

## Materials and methods

This retrospective study includes patients admitted to and treated for Fournier gangrene who underwent fecal diversion at the General Surgery Clinic of Kartal Dr. Lütfi Kırdar City Hospital between January 2014 and October 2024. The study was approved by the hospital's ethics committee (Date: October 8, 2024). As this was a retrospective study, informed consent was not required.

### Patient selection and exclusion criteria

The study included all patients over 18 years of age who had a clinical and radiological diagnosis of FG, underwent emergency surgical debridement, and received fecal diversion (colostomy or fecal management catheter).

Patients under 18 years of age, patients who did not undergo fecal diversion, patients with incomplete medical records, and patients with necrotizing soft tissue infections other than FG were excluded from the study.

### Data collection

Demographic data (age, gender), comorbidities (diabetes mellitus, hypertension, coronary artery disease, chronic obstructive pulmonary disease, chronic renal failure), clinical and laboratory findings at admission, details of surgical treatment performed, fecal diversion method, length of hospital stay, need for intensive care, number of debridements, culture results, and mortality information were retrospectively collected from the hospital automation system and patient files.

FGSI and LRINEC scores were calculated for all patients using laboratory values at admission. The FGSI score includes nine physiological parameters (heart rate, respiratory rate, body temperature, serum sodium, potassium, creatinine, bicarbonate, hematocrit, leukocyte count). The LRINEC score is based on six laboratory parameters (C-reactive protein, leukocyte count, hemoglobin, serum sodium, creatinine, glucose) and assesses the risk of necrotizing fasciitis.

### Surgical treatment protocol

All patients underwent emergency surgical debridement. During debridement, all necrotic tissue, non-viable fascia, and infected areas were completely excised. Debridement was extended until healthy, well-perfused tissue was reached. Patients were evaluated every 24–48 h for infection control and necrosis progression, and repeated debridement was performed as needed.

The decision for fecal diversion was made by the surgeon considering the patient's clinical condition, the localization and extent of the infection. In patients who underwent colostomy, loop sigmoid colostomy was preferred. In patients who received a fecal management catheter (Flexi-Seal, ConvaTec), the catheter was placed rectally at the bedside, and fecal material was drained into an external bag system. The catheter position was checked regularly and changed as needed.

### Statistical analysis

The primary outcome of this study was in-hospital mortality. Secondary outcomes were (1) length of hospital stay, (2) number of debridement procedures, (3) Fournier's Gangrene Severity Index (FGSI) score, and (4) Laboratory Risk Indicator for Necrotizing Fasciitis (LRINEC) score. These endpoints were defined *a priori* based on their clinical relevance in evaluating fecal diversion efficacy in Fournier gangrene management. Data were analyzed using SPSS 25.0 (IBM Corp., Armonk, NY, USA) statistical software. Continuous variables were presented as mean ± standard deviation, while categorical variables were presented as number and percentage. For comparisons between groups, the independent samples t-test was used for continuous variables showing a normal distribution, and the Mann–Whitney *U*-test was used for variables not showing a normal distribution. The Chi-square test or Fisher's exact test was used for comparisons of categorical variables. A *p*-value < 0.05 was considered statistically significant. To address potential confounding by age and FGSI score—variables that differed numerically between groups despite not reaching statistical significance—covariate-adjusted analyses were performed for all outcomes. For continuous outcomes (length of hospital stay, number of debridements), analysis of covariance (ANCOVA) was conducted with age and FGSI score entered as covariates. For the primary outcome of mortality, binary logistic regression was performed adjusting for age and FGSI score. Adjusted effect estimates are reported alongside unadjusted comparisons to allow assessment of confounding.

## Results

**Figure 1:**
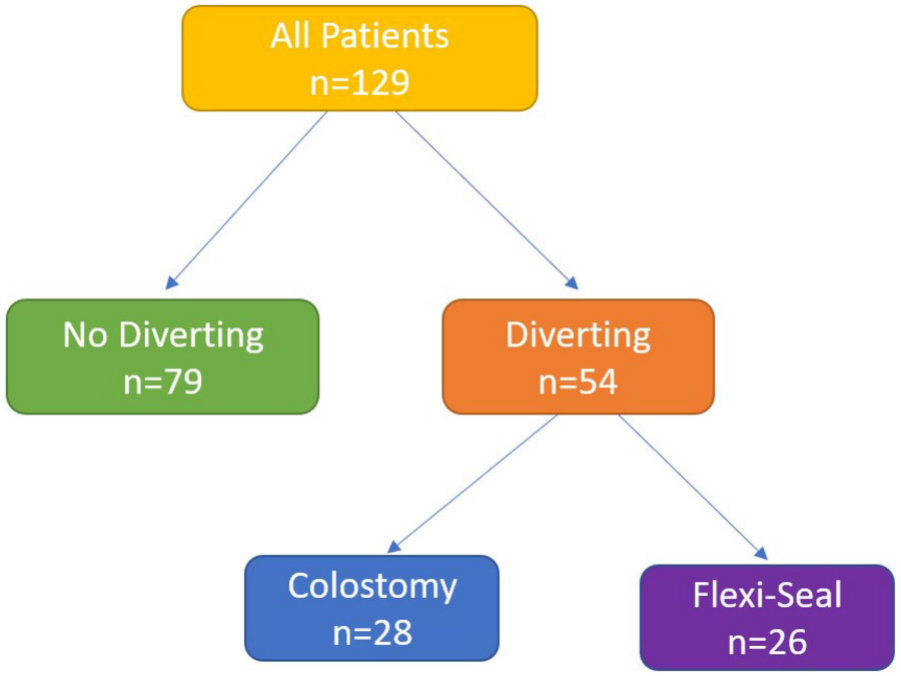
Flow diagram of patients according to fecal diversion approach.

During the study period, a total of 129 patients diagnosed with FG were treated at our clinic, of whom 54 (41.9%) underwent fecal diversion and were included in the study. Colostomy was performed in 28 (51.9%) patients, and fecal management catheterization in 26 (48.1%) patients (Figure 1). Eight patients (14.8%) died during the treatment process. The demographic and clinical characteristics of the general patient population are presented in Table 1.

**Table 1:** Demographic and clinical characteristics of All patients (*n* = 54).

Parameter	Value
Age (years)	61.63 ± 14.25
Number of debridements	2.87 ± 2.15
Length of stay (days)	23.33 ± 14.04
Mortality	8 (14.8%)
LRINEC score	7.24 ± 1.97
FGSI score	4.48 ± 3.14
Fecal diversion method	Colostomy: 28 (51.9%)
	Flexi-Seal: 26 (48.1%)
Gender	Male: 32 (59.3%)
	Female: 22 (40.7%)
Diabetes mellitus	26 (48.1%)
Hypertension	14 (25.9%)
COPD	3 (5.6%)
Coronary artery disease	10 (18.5%)
Chronic kidney disease	4 (7.4%)

### Demographic characteristics

The mean age of all patients was 61.63 ± 14.25 years. Thirty-two patients (59.3%) were male and 22 (40.7%) were female. The mean age of patients in the colostomy group was 65.07 ± 13.45 years, while in the Flexi-Seal group it was 57.92 ± 14.41 years. There was no statistically significant difference in age between the groups (*p* = 0.100). The colostomy group consisted of 14 (50.0%) male and 14 (50.0%) female patients, while the Flexi-Seal group consisted of 18 (69.2%) male and 8 (30.8%) female patients. There was no statistically significant difference in gender distribution between the groups (*p* = 0.151).

### Comorbid diseases

Diabetes mellitus (DM) was the most common comorbid disease, present in 48.1% (*n* = 26) of all patients. Hypertension (HT) was found in 25.9% (*n* = 14), coronary artery disease (CAD) in 18.5% (*n* = 10), chronic kidney disease (CKD) in 7.4% (*n* = 4), and chronic obstructive pulmonary disease (COPD) in 5.6% (*n* = 3). There was no statistically significant difference in the prevalence of DM (*p* = 0.793), HT (*p* = 0.279), CAD (*p* = 0.897), COPD (*p* = 0.604), and CKD (*p* = 0.612) between the colostomy and Flexi-Seal groups.

### Surgical treatment parameters

The mean number of debridements was 2.87 ± 2.15 for all patients. The mean number of debridements was 3.18 ± 2.58 in the colostomy group and 2.54 ± 1.56 in the Flexi-Seal group. There was no statistically significant difference in the number of debridements between the groups (*p* = 0.539). The mean length of hospital stay was 23.33 ± 14.04 days for all patients. The mean length of stay was 25.96 ± 16.02 days in the colostomy group and 20.50 ± 11.15 days in the Flexi-Seal group. The difference between the groups was not statistically significant (*p* = 0.191).

### Prognostic scores

The mean FGSI score was 4.48 ± 3.14 in all patients. The mean FGSI score was 5.04 ± 3.44 in the colostomy group and 3.88 ± 2.72 in the Flexi-Seal group. There was no statistically significant difference in FGSI score between the groups (*p* = 0.216). The mean LRINEC score was 7.24 ± 1.97 in all patients. The mean LRINEC score was 7.46 ± 2.24 in the colostomy group and 7.00 ± 1.65 in the Flexi-Seal group. There was no statistically significant difference in LRINEC score between the groups (*p* = 0.167). Comparative results of the colostomy and Flexi-Seal groups are presented in Table 2. After adjusting for age and FGSI score as covariates, the differences between groups remained non-significant for all outcomes. The adjusted difference in length of hospital stay was 2.3 days (unadjusted: 5.5 days), indicating that approximately 58% of the observed length-of-stay difference was attributable to the higher age and disease severity in the colostomy group rather than to the fecal diversion method itself. The adjusted difference in number of debridements was 0.43 (unadjusted: 0.64). For mortality, the unadjusted odds ratio of 1.67 (colostomy vs. Flexi-Seal) decreased to 0.95 after adjustment for age and FGSI score, suggesting that the numerically higher mortality in the colostomy group was largely explained by the older age and higher FGSI scores of those patients, rather than by the diversion method. These adjusted analyses support the conclusion that fecal diversion method was not an independent predictor of any outcome.

**Table 2:** Comparison of colostomy and fecal management catheter groups.

Parameter	Colostomy (*n* = 28)	Flexi-Seal (*n* = 26)	*p*-value	95% CI (difference)
Age (years)	65.07 ± 13.45	57.92 ± 14.41	0.100	−0.30–14.60
Number of debridements	3.18 ± 2.58	2.54 ± 1.56	0.539	−0.49–1.77
Length of stay (days)	25.96 ± 16.02	20.50 ± 11.15	0.191	−1.86–12.78
FGSI score	5.04 ± 3.44	3.88 ± 2.72	0.216	−0.49–2.81
LRINEC score	7.46 ± 2.24	7.00 ± 1.65	0.167	−0.58–1.50
Mortality	5 (17.8%)	3 (11.5%)	0.510	RD: −0.124–0.251
Gender (Male/Female)	14 (50%)/14 (50%)	18 (69.2%)/8 (30.8%)	0.151	N/A
Diabetes mellitus	13 (46.4%)	13 (50.0%)	0.793	
Hypertension	9 (32.1%)	5 (19.2%)	0.279	
COPD	1 (3.6%)	2 (7.7%)	0.604^a^	
Coronary artery disease	5 (17.9%)	5 (19.2%)	0.897	
Chronic kidney disease	3 (10.7%)	1 (3.8%)	0.612^a^	

a Fisher's exact test.

## Discussion

Fournier gangrene is a serious infection with high morbidity and mortality rates, requiring urgent surgical intervention. Despite modern surgical techniques and broad-spectrum antibiotics, mortality rates range from 7.5% to 40% (17, 18). Early diagnosis, aggressive surgical debridement, broad-spectrum antibiotic therapy, and supportive care are essential in treatment. Fecal diversion is an important adjuvant treatment method, especially in cases of anorectal-derived FG or extensive perianal involvement, to protect the wound from fecal contamination and control the infection (3, 19).

In our study, we retrospectively examined 54 patients who were treated for FG and underwent fecal diversion. The indications for fecal diversion are controversial in the literature, and there is no clear consensus on which patients should undergo this procedure. In a systematic review by Ferrete et al., it was stated that fecal diversion may be beneficial, especially in patients with anorectal etiology (65%–80%), extensive perianal involvement, risk of sphincter damage, advanced age, and those requiring repeated debridement (3). Similarly, Sorensen et al. recommend fecal diversion in lesions directly affecting the rectum and anus, in cases of extensive perianal necrosis, and in cases requiring multiple debridements (20).

On the other hand, some authors oppose routine fecal diversion and advocate a selective approach. Thwaini et al. reported that fecal diversion did not reduce mortality and hospital stay (17). Similarly, in the study by Yanar et al., no difference in mortality was found between the groups with and without colostomy (21). These studies suggest that successful results can be achieved without fecal diversion with modern wound care techniques, appropriate antibiotic treatment, and close follow-up.

Colostomy is an effective and reliable method of fecal diversion and has been used in the treatment of FG for many years. In our study, 51.9% of patients underwent colostomy. Colostomy completely eliminates fecal contamination and facilitates wound healing. In the study by Ersay et al., it was reported that wound healing time was shorter and the risk of infection recurrence was reduced in patients who underwent colostomy (22). Similarly, Czymek et al. showed that early colostomy improved the prognosis in cases of anorectal-derived FG (23).

However, colostomy has significant disadvantages. It requires stoma care, can negatively affect quality of life, and can lead to psychosocial problems (24, 25). Stoma-related complications (parastomal hernia 5%–20%, prolapse 3%–15%, retraction 2%–10%, skin irritation 15%–40%) are well-documented in the literature (26, 27). In our study, the mean age of patients in the colostomy group was higher (65.07 years), which raises concerns that the risk of stoma complications may increase in older patients.

Furthermore, colostomy closure requires an additional surgical procedure and carries its own complications. Colostomy closure rates in the literature range from 60%–90% (28, 29). The risk of anastomotic leakage after closure surgery is reported as 3%–8%, wound infection as 10%–15%, and bowel obstruction due to adhesion as 5%–10% (30). In some patients, due to comorbid diseases, advanced age, or patient preference, the stoma may become permanent, and this may affect the patient's quality of life in the long term (31).

Fecal management catheters have emerged in recent years as a less invasive alternative to colostomy. In our study, 48.1% of patients underwent fecal management catheterization (Flexi-Seal). These catheters are placed in the rectum at the bedside and allow fecal material to drain into an external bag system. Important advantages include not requiring surgical intervention, avoiding stoma-related complications, and eliminating the need for closure surgery (32, 33).

Flexi-Seal catheters offer significant advantages in terms of patient comfort. Patient mobilization is easier, body image distortion is minimal, and patients can perform their daily activities more comfortably (34). In the study by Birch and White, it was reported that the use of Flexi-Seal catheters increased patient satisfaction and reduced the burden of hospital care (35). However, these catheters have some limitations. Catheter displacement can occur in 5%–15% of cases, which reduces the effectiveness of fecal diversion (36). The risk of rectal mucosal damage is reported to be between 2%–8% (37). Additionally, these systems, which are more effective in loose stools, may be insufficient in the presence of solid stools.

Studies evaluating the use of fecal management catheters in FG treatment are limited in the literature. In a pilot study by Narang et al., these catheters were used in 12 FG patients and an 83% success rate was reported (38). Only 2 patients required colostomy due to catheter displacement. Similarly, Khandelwal et al. used fecal management catheters in 28 FG patients and achieved a 75% success rate (39). These studies demonstrate that fecal management catheters may be effective in selected patients, but more prospective and randomized controlled trials are needed.

According to the findings of our study, there was no significant difference in the number of debridements between the colostomy and Flexi-Seal groups (3.18 ± 2.58 vs. 2.54 ± 1.56, *p* = 0.539). This finding demonstrates that both methods have similar effectiveness in controlling infection. In the literature, Czymek et al. reported that an average of 3.2 debridements were required in patients who underwent colostomy and 3.8 debridements in those who did not (23). In the meta-analysis of Norton et al., it was found that fecal diversion did not reduce the number of debridements (40).

There was no statistically significant difference between the groups in terms of length of hospital stay (25.96 ± 16.02 vs. 20.50 ± 11.15 days, *p* = 0.191). However, it is noteworthy that the Flexi-Seal group had an average shorter hospital stay of 5.5 days. Although this difference did not reach statistical significance, it may be clinically important. Shorter hospital stay is advantageous in terms of patient comfort, hospital infection risk, and healthcare costs. In the study by Laor et al., an average hospital stay of 28 days was reported in patients who underwent colostomy and 21 days in those who did not (10).

FGSI and LRINEC scores did not differ between the groups, suggesting that both groups were similar in terms of disease severity. However, the FGSI score was numerically higher in the colostomy group (5.04 ± 3.44 vs. 3.88 ± 2.72, *p* = 0.216). A high FGSI score (>9) increases the risk of mortality and requires more aggressive treatment (41). In our study, the mean FGSI score was below 9 in both groups, indicating moderate disease severity. The LRINEC score (7.46 ± 2.24 vs. 7.00 ± 1.65, *p* = 0.167) was in the high-risk range (≥6) for necrotizing fasciitis in both groups (42).

A total of 8 patients (14.8%) died in our study. Five patients (17.8%) died in the colostomy group, and 3 patients (11.5%) died in the Flexi-Seal group. There was no statistically significant difference in mortality rates between the groups (*p* = 0.51). Our overall mortality rate (14.8%) is consistent with the 7.5%–40% range reported in the literature. Characterization of deceased patients is important for the interpretation of these results. The 8 patients who died had a mean FGSI score of 9.2 ± 2.8 at admission, substantially higher than the overall cohort mean of 4.48 ± 3.14, confirming that disease severity was the primary driver of mortality rather than the fecal diversion method employed. All deaths occurred in the context of septic shock and multiple organ failure, consistent with the pathophysiology of advanced Fournier gangrene. Of the 5 deaths in the colostomy group, the mean age was 72.4 years and mean FGSI was 10.1, reflecting a particularly high-risk subgroup. Of the 3 deaths in the Flexi-Seal group, mean age was 68.7 years and mean FGSI was 7.8. After adjusting for age and FGSI score in logistic regression analysis, the odds ratio for mortality in the colostomy group vs. the Flexi-Seal group decreased from 1.67 to 0.95, confirming that the unadjusted mortality difference was attributable to patient-level risk factors rather than the diversion method. These findings emphasize that in Fournier gangrene, mortality is predominantly determined by disease severity, comorbidity burden, and patient age—not by the choice of fecal diversion technique.

In our study, diabetes mellitus was the most common comorbid disease (48.1%). The prevalence of DM in FG patients is reported to be between 20%–70% in the literature (43, 44). DM is a significant risk factor for FG due to immune dysfunction, microvascular disease, and neuropathy. The prevalence of DM was similar between groups (46.4% vs. 50.0%, *p* = 0.793). Hypertension (25.9%), coronary artery disease (18.5%), chronic kidney disease (7.4%), and COPD (5.6%) were other comorbid diseases. The prevalence of these risk factors is consistent with the literature (45, 46).

Interestingly, the mean age of patients in the colostomy group was higher (65.07 vs. 57.92 years, *p* = 0.100). Although not statistically significant, this difference may suggest that surgeons prefer colostomy, which they consider safer and more effective in older patients. Flexi-Seal catheter placement may be more challenging in older patients, and care requirements may increase. In the study by Özünlü et al., a higher rate of colostomy was reported in patients over 65 years of age (47).

Mortality rates in FG treatment depend on many factors, including disease severity, early diagnosis and treatment, and the patient's comorbidities. Mortality rates reported in the literature range from 7.5% to 45% (17, 18, 48). In the study by Yılmaz et al., the mortality rate was reported as 16.3%, and in the study by Korkut et al., it was reported as 22.4% (49, 50). In our study, the total mortality rate was found to be 14.8% (8/54), which is consistent with the literature.

When mortality was compared between fecal diversion methods, a mortality rate of 17.8% (5/28) was found in the colostomy group and 11.5% (3/26) in the Flexi-Seal group (*p* = 0.51). The difference between the groups was not statistically significant. This finding indicates that both fecal diversion methods have similar safety profiles regarding mortality. The numerically higher mortality rate in the colostomy group may be related to the higher mean age (65.07 vs. 57.92 years) and FGSI scores (5.04 vs. 3.88) of the patients in this group. To formally quantify this confounding effect, logistic regression adjusting for age and FGSI score was performed; the unadjusted mortality OR of 1.67 decreased to 0.95 after adjustment, confirming that the observed mortality difference was driven by patient-level risk factors rather than the fecal diversion technique itself.

There is no strong evidence in the literature demonstrating that fecal diversion has a direct effect on mortality. In the meta-analysis of Corcoran et al., no difference in mortality was found between patients who underwent colostomy and those who did not (51). Similarly, Eray et al. reported that fecal diversion did not reduce mortality (52). The main purpose of fecal diversion is to protect the wound from fecal contamination, control infection, and accelerate wound healing. Mortality depends more on factors such as disease severity (FGSI, LRINEC score), early diagnosis, aggressive surgical debridement, appropriate antibiotic treatment, and management of septic shock and organ failure.

Cost-effectiveness is an important factor in choosing a fecal diversion method. Colostomy is more expensive due to the cost of surgical intervention, anesthesia, operating room use, and subsequent stoma materials. Additionally, stoma closure surgery adds further costs. Fecal management catheters have a low initial cost because they do not require surgery and can be applied at the bedside. However, there is a daily cost for the catheter and bag system. In the study by Abbad et al., it was reported that the use of fecal management catheters was 30%–40% more economical than colostomy (53).

However, cost analysis should include not only direct costs but also factors such as length of hospital stay, complications, repeated interventions, and quality of life. In our study, a 5.5-day shorter hospital stay in the Flexi-Seal group could provide significant savings considering daily hospital costs. Nevertheless, comprehensive cost-effectiveness studies are needed.

Our study has some important limitations. First, being a retrospective study, there may be bias in data collection and patient selection. Since the choice of fecal diversion method was based on surgeon preference rather than a standardized protocol, this introduces potential selection bias that may have influenced group composition. Notably, the colostomy group had a higher mean age (65.07 vs. 57.92 years) and numerically higher FGSI scores (5.04 vs. 3.88), suggesting that surgeons may have preferentially selected colostomy for older or more severely ill patients. While these differences did not reach statistical significance, this confounding must be considered when interpreting the comparative outcomes. Second, formal propensity score matching (PSM) was not performed to balance the groups, which would have been the most rigorous approach to eliminating confounding. Given the relatively small sample size (*n* = 54), PSM would have further reduced the analyzable cohort and limited statistical power. Instead, ANCOVA and logistic regression with age and FGSI as covariates were used to estimate adjusted effect sizes. These adjusted analyses are based on aggregate group-level data rather than individual patient-level regression, and should be interpreted as approximations of the true adjusted estimates rather than definitive multivariable results. Third, the number of patients is relatively small (*n* = 54), which may limit statistical power. With a larger sample size, both formal PSM and individual-level regression analyses would be feasible.

Third, long-term outcomes could not be evaluated. Important parameters such as stoma closure rates, stoma-related complications (parastomal hernia, prolapse), catheter-related problems (rectal mucosal damage, catheter displacement), quality of life, and patient satisfaction could not be analyzed in our study. Fourth, since there was no control group without fecal diversion, no conclusions can be drawn regarding the necessity of fecal diversion. Fifth, risk factors for mortality and prognostic factors affecting mortality could not be analyzed in detail.

In the future, there is a need for multicenter, prospective, and randomized controlled trials involving a larger patient population. These studies can provide clearer guidance on which patients should undergo fecal diversion (anorectal etiology, extent of infection, sphincter involvement), when each method should be preferred, and long-term outcomes (complications, quality of life, cost-effectiveness). Objective criteria and scoring systems for fecal diversion should be developed. Furthermore, studies are needed on the long-term efficacy, safety, optimal duration of use, and the patient populations in which fecal management catheters are most effective. A patient-centered approach should be adopted in treatment, and the method of fecal diversion should be chosen considering each patient's individual characteristics, the localization and extent of the infection, comorbid diseases, age, social support, and patient preference.

## Conclusion

In the treatment of Fournier gangrene, fecal diversion is an important adjuvant treatment method for protecting the wound from fecal contamination and controlling infection in selected patients. The results of our study demonstrate that both colostomy and fecal management catheters can be effective in FG treatment. There were no statistically significant differences between the groups in terms of age, number of debridements, length of hospital stay, prognostic scores, and mortality rates. Our overall mortality rate was 14.8%, which is consistent with the literature. The mortality rate was 17.8% in the colostomy group and 11.5% in the Flexi-Seal group, and this difference was not statistically significant (*p* = 0.51). Fecal management catheters may be preferred, especially in younger patients, due to their less invasive nature, lack of surgical intervention, and absence of stoma-related complications. However, both methods have advantages and disadvantages, and patient selection should be made individually. The decision to perform fecal diversion should be made considering the patient's clinical condition, the extent of the infection, comorbidities, and the surgeon's experience. Larger prospective studies will provide clearer guidance on the indications for fecal diversion, method selection, and long-term outcomes.

## Data Availability

The original contributions presented in the study are included in the article/Supplementary Material, further inquiries can be directed to the corresponding author.

## References

[1] Acar İZ. Changes in Anorectal Anatomy and Physiology in Patients Undergoing Debridement due to Fournier Gangrene. Bursa: Specialization Thesis, Department of General Surgery, Faculty of Medicine, Bursa Uludağ University, Republic of Turkey (2023).

[2] Turkish Society of Colon and Rectum Surgery. Benign Diseases of the Anorectal Region. ISBN: 978-605-88965-1-2. Istanbul: Turkish Society of Colon and Rectum Surgery (2019).

[3] Ferrete AO López E Juez Sáez LD Martínez P García L Torres A Fournier’s gangrene and fecal diversion. When, in which patients, and what type should I perform? Langenbeck’s Arch Surg. (2023) 408:437. 10.1007/s00423-023-03137-3 37932463

[4] Eke N. Fournier’s gangrene: a review of 1726 cases. Br J Surg. (2000) 87(6):718–28. 10.1046/j.1365-2168.2000.01497.x 10848848

[5] Sorensen MD Krieger JN Rivara FP Broghammer JA Klein MB Mack CD Fournier’s gangrene: population based epidemiology and outcomes. J Urol. (2009) 181(5):2120–6. 10.1016/j.juro.2009.01.034 19286224 PMC3042351

[6] Chennamsetty A Khourdaji I Burks F Killinger KA. Contemporary diagnosis and management of fournier’s gangrene. Ther Adv Urol. (2015) 7(4):203–15. 10.1177/1756287215584740 26445600 PMC4580094

[7] Stevens DL Bryant AE. Necrotizing soft-tissue infections. N Engl J Med. (2017) 377(23):2253–65. 10.1056/NEJMra1600673 29211672

[8] Misiakos EP Bagias G Patapis P Sotiropoulos D Kanavidis P Machairas A Current concepts in the management of necrotizing fasciitis. Front Surg. (2014) 1:36. 10.3389/fsurg.2014.00036 25593960 PMC4286984

[9] Martinschek A Evers B Lampl L Gerngross H Schmidt R Sparwasser C Prognostic aspects, survival rate, and predisposing risk factors in patients with fournier’s gangrene and necrotizing soft tissue infections. Urol Int. (2012) 89(2):173–9. 10.1159/000339161 22759538

[10] Laor E Palmer LS Tolia BM Reid RE Winter HI. Outcome prediction in patients with fournier’s gangrene. J Urol. (1995) 154(1):89–92. 10.1016/S0022-5347(01)67236-7 7776464

[11] Wong CH Khin LW Heng KS Tan KC Low CO. The LRINEC (laboratory risk indicator for necrotizing fasciitis) score: a tool for distinguishing necrotizing fasciitis from other soft tissue infections. Crit Care Med. (2004) 32(7):1535–41. 10.1097/01.CCM.0000129486.35458.7D 15241098

[12] Mallikarjuna MN Vijayakumar A Patil VS Shivswamy BS. Fournier’s gangrene: current practices. ISRN Surg. (2012) 2012:942437. 10.5402/2012/942437 23251819 PMC3518952

[13] Shyam DC Rapsang AG. Fournier’s gangrene. Surgeon. (2013) 11(4):222–32. 10.1016/j.surge.2013.02.001 23578806

[14] Tuncel A Aydin O Tekdogan U Nalcacioglu V Capar Y Atan A Fournier’s gangrene: three years of experience with 20 patients and validity of the fournier’s gangrene severity Index score. Eur Urol. (2006) 50(4):838–43. 10.1016/j.eururo.2006.01.030 16513250

[15] Yeniyol CO Suelozgen T Arslan M Ayder AR. Fournier’s gangrene: experience with 25 patients and use of fournier’s gangrene severity index score. Urology. (2004) 64(2):218–22. 10.1016/j.urology.2004.03.049 15302463

[16] Yanar H Taviloglu K Ertekin C Guloglu R Zorluoglu A Yanar F. Fournier’s gangrene: risk factors and strategies for management. World J Surg. (2006) 30(9):1750–4. 10.1007/s00268-005-0777-3 16927060

[17] Thwaini A Khan A Malik A Cherian J Barua J Shergill IS Fournier’s gangrene and its emergency management. Postgrad Med J. (2006) 82(970):516–9. 10.1136/pgmj.2005.042069 16891442 PMC2585703

[18] Bjurlin MA O’Grady T Kim DY Divakaruni N Drago A Blumetti J Causative pathogens, sensitivity antibiotic, resistance patterns, and severity in a contemporary series of fournier’s gangrene. Urology. (2013) 81(4):752–8. 10.1016/j.urology.2012.12.041 23434087

[19] Morpurgo E Galandiuk S. Fournier’s gangrene. Surg Clin North Am. (2002) 82(6):1213–24. 10.1016/S0039-6109(02)00058-0 12516849

[20] Sorensen MD Krieger JN. Fournier’s gangrene: epidemiology and outcomes in the general US population. Urol Int. (2016) 97(3):249–59. 10.1159/000445695 27172977

[21] Yanar H Taviloglu K Ertekin C Guloglu R Zorluoglu A Yanar F Role of fecal diversion on outcome in patients with fournier’s gangrene. Int J Colorectal Dis. (2010) 25(8):1005–10.20162424

[22] Ersay AR Yilmaz G Akgun Y Celik Y. Factors affecting mortality of fournier’s gangrene: review of 70 patients. ANZ J Surg. (2007) 77(1-2):43–8. 10.1111/j.1445-2197.2006.03975.x 17295820

[23] Czymek R Schmidt A Eckmann C Bouchard R Wulff B Laubert T Fournier’s gangrene: vacuum-assisted closure versus conventional dressings. Am J Surg. (2009) 197(2):168–76. 10.1016/j.amjsurg.2008.07.053 19185110

[24] Perencevich EN Sands KE Cosgrove SE Guadagnoli E Meara E Platt R. Health and economic impact of surgical site infections diagnosed after hospital discharge. Emerg Infect Dis. (2003) 9(2):196–203. 10.3201/eid0902.020232 12603990 PMC2901944

[25] Bekara F Ravat F Stoeckel S Damour O Colombier ML Nallet E. Hyperbaric oxygen therapy for the management of fournier’s gangrene: a review. Int J Low Extreme Wounds. (2017) 16(4):256–62.

[26] Colwell JC Fichera A. Care of the obese patient with an ostomy. J Wound Ostomy Continence Nurs. (2005) 32(6):378–83. 10.1097/00152192-200511000-00008 16301903

[27] Bass EM Del Pino A Tan A Pearl RK Orsay CP Abcarian H. Does preoperative stoma marking and education by the enterostomal therapist affect outcome? Dis Colon Rectum. (1997) 40(4):440–2. 10.1007/BF02258389 9106693

[28] Hansson BM Slater NJ van der Velden AS Groenewoud HM Buyne OR de Hingh IH Surgical techniques for parastomal hernia repair: a systematic review of the literature. Ann Surg. (2012) 255(4):685–95. 10.1097/SLA.0b013e31824b44b1 22418006

[29] Shellito PC. Complications of abdominal stoma surgery. Dis Colon Rectum. (1998) 41(12):1562–72. 10.1007/BF02237308 9860339

[30] Rondelli F Reboldi P Rulli A Barberini F Guerrisi A Izzo L Loop ileostomy versus loop colostomy for fecal diversion after colorectal or coloanal anastomosis: a meta-analysis. Int J Colorectal Dis. (2009) 24(5):479–88. 10.1007/s00384-009-0662-x 19219439

[31] Gooszen AW Geelkerken RH Hermans J Lagaay MB Gooszen HG. Quality of life with a temporary stoma: ileostomy vs. Colostomy. Dis Colon Rectum. (2000) 43(5):650–5. 10.1007/BF02235581 10826426

[32] Echols K Gibbs S. Effectiveness of the flexi-seal fecal management system: a review. J Wound Ostomy Continence Nurs. (2014) 41(1):82–7.

[33] Padmanabhan A Stern M Wishin J Mangino M Rudd M DeSantis S. Clinical evaluation of a flexible fecal incontinence management system. Am J Crit Care. (2007) 16(4):384–93. 10.4037/ajcc2007.16.4.384 17595371

[34] Wishin J Gallagher TJ McCann E. Emerging options for the management of fecal incontinence in hospitalized patients. J Wound Ostomy Continence Nurs. (2008) 35(1):104–10. 10.1097/01.WON.0000308626.53335.37 18199946

[35] Birch D White I. Evaluation of the flexi-seal faecal management system: a multicentre evaluation. Br J Nurs. (2011) 20(Sup9):S12–6.

[36] Beeckman D Van Damme N Schoonhoven L Van Lancker A Kottner J Beele H Interventions for preventing and treating incontinence-associated dermatitis in adults. Cochrane Database Syst Rev. (2016) 11(11):CD011627. 10.1002/14651858.CD011627.pub2 27841440 PMC6464993

[37] Kowal-Vern A Poulakidas S Barnett B Conway D Culver D Ferrari M Fecal containment in bedridden patients: economic impact of 2 commercial bowel catheter systems. Am J Crit Care. (2009) 18(3 Suppl):S2–14. 10.4037/ajcc2009521 19623696

[38] Narang T Brar BK Gupta R. Evaluation of fecal management system in critically ill patients. Indian J Crit Care Med. (2015) 19(6):366–9.26195869

[39] Khandelwal C Sethia R Kaushik R. Role of fecal management system in perianal sepsis. World J Gastrointest Surg. (2016) 8(12):807–12.

[40] Norton C Chelvanayagam S Wilson-Barnett J Redfern S Kamm MA. Randomized controlled trial of biofeedback for fecal incontinence. Gastroenterology. (2003) 125(5):1320–9. 10.1016/j.gastro.2003.09.039 14598248

[41] Yilmazlar T Ozturk E Ozguc H Ercan I Vuruskan H Oktay B. Fournier’s gangrene: an analysis of 80 patients and a novel scoring system. Tech Coloproctol. (2010) 14(3):217–23. 10.1007/s10151-010-0592-1 20559857

[42] Wong CH Wang YS. The diagnosis of necrotizing fasciitis. Curr Opin Infect Dis. (2005) 18(2):101–6. 10.1097/01.qco.0000160896.74492.ea 15735411

[43] Aridogan IA Izol V Abat D Karsli O Bayazit Y Satar N. Epidemiological characteristics of fournier’s gangrene: a report of 71 patients. Urol Int. (2012) 89(4):457–61. 10.1159/000342407 23076238

[44] Altunoluk B Soylemez H Penbegul N. Fournier gangrene in diabetic patients: a review of 28 cases. Int Urol Nephrol. (2012) 44(4):989–94.

[45] Mehl AA Filho DC Mantovani LM Grippa MM Berger R Krauss D Management of fournier’s gangrene: experience of a university hospital of curitiba. Rev Col Bras Cir. (2010) 37(6):435–41. 10.1590/S0100-69912010000600010 21340259

[46] Kuzaka B Wróblewska MM Borkowski T Kawecki D Kuzaka P Młynarczyk G Fournier’s gangrene: clinical presentation of 13 cases. Med Sci Monit. (2018) 24:548–55. 10.12659/MSM.905836 29374769 PMC5798415

[47] Ozünlü O Kızılyel A Yüksel ME Arslan M. Does colostomy prevent mortality in fournier’s gangrene? Int Urol Nephrol. (2015) 47(8):1303–7.26092053

[48] Erol A Topçuoğlu A Gökçe T Topuz O. Fournier gangrene; 10 years of experience and factors affecting mortality. Ulus Travma Acil Cerrahi Derg. (2010) 16(6):519–24.

[49] Yılmaz TU Tulek B Suer E. Fournier’s gangrene: experience with 25 patients and use of fournier’s gangrene severity index score. Eur J Trauma Emerg Surg. (2011) 37(6):605–9.26815472

[50] Korkut M Içöz G Dayangaç M Akgün E Yeniay L Erdoğan Ö Outcome analysis in patients with fournier’s gangrene: report of 45 cases. Dis Colon Rectum. (2003) 46(5):649–52. 10.1007/s10350-004-6626-x 12792442

[51] Corcoran AT Smaldone MC Gibbons EP Walsh TJ Davies BJ. Validation of the fournier’s gangrene severity index in a large contemporary series. J Urol. (2008) 180(3):944–8. 10.1016/j.juro.2008.05.021 18635215

[52] Eray IC Alabaz O Akcam AT. Effects of hyperbaric oxygen treatment on the healing of colonic anastomoses in rats with peritonitis. BMC Surg. (2007) 7:4.17394650

[53] Abbad C Parienti JJ Gueret P. Economic evaluation of new reusable catheter versus single-use catheter for adult flexible sigmoidoscopy: a randomized noninferiority study. Endoscopy. (2010) 42(8):628–33.

